# Rethinking the Social Determination of Food in Chile Through Practices and Interactions of Actors in Food Environments: Nonexperimental, Cross-Sectional Study

**DOI:** 10.2196/62765

**Published:** 2024-09-13

**Authors:** Patricia Gálvez Espinoza, Lorena Rodríguez Osiac, Carolina Franch Maggiolo, Daniel Egaña Rojas

**Affiliations:** 1 Department of Nutrition Universidad de Chile Santiago Chile; 2 School of Public Health Universidad de Chile Santiago Chile; 3 Department of Anthropology Universidad de Chile Santiago Chile; 4 Department of Primary Care and Family Health Universidad de Chile Santiago Chile

**Keywords:** mixed methods, research design, food environments, food intake, obesity

## Abstract

**Background:**

Food environments are crucial for promoting healthy and sustainable eating and preventing obesity. However, existing food environment frameworks assume an already installed causality and do not explain how associations in food environments are established or articulated, especially from an integrative and transdisciplinary approach. This research attempts to bridge these gaps through the use of Actor-Network Theory, which traces the relationship network between human (and nonhuman) actors in order to describe how these interact and what agencies (direct or remote) are involved.

**Objective:**

This study aims to explain the practices and interactions of actors in food environments in order to approach the problem of unhealthy eating with a transdisciplinary approach.

**Methods:**

This is a nonexperimental, cross-sectional study. Due to the complexity of the study phenomena, a mixed methods approach with 4 consecutive phases will be developed in Chile. Phase 1 involves a systematic literature review of food environment evidence since 2015, following the PRISMA (Preferred Reporting Items for Systematic Reviews and Meta-Analyses) protocol; phase 2 involves the application of a shortened version of the Nutrition Environment Measure Scale—Perceptions adapted to Chile (NEMS-P-Ch) in 2 neighborhoods with different socioeconomic levels; in phase 3, six focus groups in each neighborhood will be conducted to address social determinants such as gender, employment status, and migration; and in phase 4, participant observation and in-depth interviews will be used to analyze the direct and empirical exploration of the actors in their daily interaction with food environments. The triangulation and complementarity of the data will allow us to create a practical model about the practices and interactions of actors in their food environments, which reflects the complexity and transdisciplinary nature of the study.

**Results:**

We have advanced in phases 1-3 of the study. In phase 1, a total of 109 manuscripts are being revised for data extraction. In phase 2, we applied the NEMS-P-Ch to 785 people, 49.4% (388/785) of whom belong to a low socioeconomic neighborhood. Participants from phase 2 are being contacted to participate in the focus groups (phase 3). By the end of July, we have conducted 6 focus groups with 5-11 participants.

**Conclusions:**

This study will provide a comprehensive understanding of how individuals interact with their food environments, offering deep insights into the factors influencing their food-related decisions. In addition, the study aims to develop a model that more accurately reflects reality by examining not only the food environments themselves but also the interactions among various stakeholders within these environments and their daily practices. The findings of this study will offer evidence-based insights to inform public policies tailored to the specific territories and communities under investigation or those with similar characteristics.

**International Registered Report Identifier (IRRID):**

DERR1-10.2196/62765

## Introduction

Unhealthy eating, characterized by a high intake of ultraprocessed foods (such as high-sugar foods and drinks and foods high in saturated and trans fats) and a low intake of fiber-rich foods (such as fruits, vegetables, and legumes), is a global public health challenge due to its association with obesity and noncommunicable diseases [1,2]. According to the World Health Organization, “three billion people cannot access safe, nutritious, and sufficient food” [2]. Data from the Global Nutrition Report indicate that people have a low intake of vegetables (40% below recommendation), fruits (60% below recommendation), whole grains (61% below recommendation), and legumes and nuts (between 68% and 74% of recommendation) [3]. However, red meat intake is more than 300% above the recommended amount [3]. Ultraprocessed foods represent between 50% and 60% of the daily energy that people consume, meaning that natural or less processed foods are being replaced by these foods [4,5].

Using a comparative risk assessment of 195 countries, the GBD (Global Burden of Diseases, Injuries, and Risk Factors Study) 2017 Diet Collaborators [6] discovered that dietary risk factors were responsible for 11 million deaths and 255 million disability-adjusted life-years in 2017. A recent review and meta-analysis showed that high exposure to ultraprocessed foods is associated with an increased risk of adverse health outcomes, such as cardiometabolic and mental disorders and mortality [7]. Addressing and solving the problem of an unhealthy diet requires a transdisciplinary approach, which goes beyond the biomedical or unidisciplinary methods that tend to oversimplify it.

The evidence indicates that environmental factors are the most relevant elements to explain an unhealthy diet and its consequences [8,9]. An individual will be able to perform some behaviors (eg, eat more fruits and vegetables) only if his or her environment is conducive to those behaviors (eg, if fruits and vegetables are available nearby and at affordable prices) [10-12]. This calls for conceptualizing food environments, of which several definitions exist [13,14]. One of the most recent and comprehensive is contained in the report written by the expert panel on food security and nutrition of the Committee on World Food Security, which defines food environments as *the physical, economic, political, and sociocultural context that frames consumers’ interaction with the food system for the acquisition, preparation, and consumption of foods* [15]. This environment is formed by its physical configuration, sociocultural rules, networks of influence and determinations of public and private stakeholders, and the socioeconomic level [14]. It has also been established that food environments are essential for promoting the consumption of healthy and sustainable food, as well as for obesity prevention and improvements in people’s quality of life [13,15,16].

The problem with the current food environment is that people are surrounded by obesogenic food environments, which are defined as the ones that bring together opportunities and conditions to promote unhealthy behavior patterns, thus leading to the accumulation of body fat in individuals and obesity in populations [17]. These obesogenic environments predominate in territories with lower socioeconomic levels, establishing unacceptable inequalities that are directly related to the differences in the prevalence of obesity among individuals and communities [18,19]. A recent literature review and meta-analysis showed that living in low socioeconomic neighborhoods increases the probability of being overweight by 31% (odds ratio 1.45, 95% CI 1.21-1.74; *P*<.001) and of being obese by 45% (odds ratio 1.31, 95% CI 1.16-1.47; *P*<.001), compared with high socioeconomic neighborhoods [20].

Chile has been fighting against unhealthy diet and obesity in its population for decades through several public policies [21]. Still, the latest National Health Survey 2016-2017 found that just 15% of the population met the recommendation of 5 servings of fruits and vegetables daily, 9.2% eat fish or other types of seafood, and 24.4% consume legumes at least twice a week [22]. According to the Pan American Health Organization, Chile is one of the countries with the highest sales of ultraprocessed foods in Latin America [16]. In addition, more than 85% of the population is sedentary. As a consequence, the prevalence of overweight, obesity, and morbid obesity affects 74.2% of the population. These conditions affect people’s full development and their quality of life, decreasing the years of life that are free from disease and disability and increasing early mortality [23]. This context generates the need to seek new research and work approaches that provide a more comprehensive view, that is, one that goes beyond the traditional limits of only recommending specific behavior changes to people.

In 2016, Cerda et al [24] proposed a model of food environments for Chile through a set of definitions that propose 5 environments: domestic, institutional, street, restaurants (including restaurants, coffee shops, or similar), and supply. In this model, the domestic food environment is considered the starting point for the mobilization of the subjects to other environments. It also states that environments interact with each other and are crossed by cultural, economic, and social dimensions [24,25]. This model of food environments has served as a framework for Chilean policies such as the National Food and Nutrition Policy [26] and the National Health Strategy to establish the health objectives for the decade until 2030 [27].

An interesting aspect of this model is that it breaks with the usual barriers of individual approaches and forces the integration of disciplines to understand the problem [28]. It also makes it possible to problematize actions in specific environments that are permeable to the daily routines of individuals and communities. In addition, the model includes cultural and social dimensions, which have a great influence on food preferences and practices, complicating the idea that people can “choose to live healthy” when, in fact, lifestyle choices are the result of a complex interaction between all the determining factors (particularly the food environment, the socioeconomic level, and cultural factors). The environment in which we live has a series of “traps” that make it difficult to maintain a healthy life, even when we know which behaviors are desirable [29].

Although there is evidence on some of the food environments in Chile, there are still some problems to be addressed so as to better understand how to determine these environments when analyzing people’s nutritional health. Most studies continue to analyze environments associated with a more or less direct response variable (eg, BMI, food availability), although evidence shows that environmental influences on diet are complex, relational, and dynamic [30]. Generally, conceptual frameworks (including those related to social determinants of food and food environments) assume an already established causality while describing structural determinants that limit and restrict people’s practices; thus, they do not explain how these associations are established or how they interact with each other. These frameworks also fail to explain how these determinations are embodied and structured in the practices of daily life, which contributes to the lack of understanding of, for example, those “traps that make it difficult to lead a healthy life.” As the sociologist of science Bruno Latour [31] contends, these conceptual frameworks are assembled versions of the social aspect. Most of the work that has been done on food environments, according to Thompson et al [32], adopts a “black box” approach because it neither explores how residents of disadvantaged neighborhoods respond to their neighborhood’s food environment nor what shapes these different responses beyond better or worse access to a wider range of foods. Turner et al [33] argue that in food environment research, the emphasis on interactions contributes to anchoring the framework of the food environment within the context of individuals’ daily routines and behaviors, which influence their dietary habits, adding a more comprehensive point of view.

Moreover, the emergence of digital technology (especially during the COVID-19 pandemic) makes it necessary to reconsider the definitions of food environments, as this technology creates new ways of interacting *with* and *between* different environments. For instance, digital technology allows food sales and purchases, changes food availability, and ignores physical distance and time spent shopping, all while increasing marketing exposure by influencing choices, preferences, and food consumption [34].

These new approaches can be investigated through theoretical perspectives from social science. In the 1970s and 1980s, Actor-Network Theory (ANT) emerged from the work of Latour, Law, and Carron, who questioned the social models that conceal the type of associative dynamics of human (and nonhuman) actors (actants) [35]. ANT attempts to trace the network of relationships between these actors (objects, technology, ideas, devices, animals, vegetables, etc) in order to describe how they interact and which agencies (direct or remote) are involved [31]. This is possible through the traces left by relationships and interactions; however, it is not limited to those produced by human agents, as symmetrically, it also considers the traces produced by the environment itself and its objects [35]. According to Oña Serrano and Viteri Salazar [36], as a conceptual, methodological proposal, ANT would be particularly productive for addressing the complexity of the food system (including the food environment), its interactions, and the multiple economic, sociocultural, and internal and external environmental actors, allowing for a better understanding of the relationships between humans and nonhumans [35]. For Goodman [37], ANT allows food studies to be reframed by problematizing the ontological dichotomy between nature (nonhuman) and society. Following Latour [38], ANT proposes a framework in which interactions are conceptualized in terms of heterogeneous collective associations between elements of nature and elements of the social world, which are assembled in networks of actors. In this line, the agency is collective and relational, as well as a consequence of these hybrid networks between human and nonhuman actors.

Unlike social models that propose “coldly” assembled causal and deterministic structural relationships, ANT proposes the tracking of the actors when the relationships they establish with their environment are more unstable and “hot” [39]. For ANT, an actant (regardless of whether it is human or not) is so when it operates as a mediator for another actant. On the other hand, *tracking* the actors means following the rhizomatic network they build from their interactions with other actors (eg, humans, objects, food, institutions, and speeches, among others) and paying attention to how “they compose the social without previously imposing order on them” [31].

There is already some use of ANT in food studies. de Sowa and Busch [40] researched soy production and consumption in Brazil; Busch and Juska [41] analyzed global food system networks; and Lockie [42] used ANT to trace the interactions between organic food consumers in Australia and a holistic approach to the agri-food system. Most recently, Legun [43] followed an entire process ranging from apple production to their consumption to reflect on the role of technology in shaping food markets, while Stoddard and Cantor [44] used ANT to trace the web of vulnerability around the pork industry in North Carolina.

In this context, food environments represent a privileged space for monitoring networks and relationships between different actors, including those within the food environments themselves. Understanding these dynamics will contribute to comprehending the influence of food environments on diets and people’s lives. It is necessary to understand these complex interactions of actors within food environments to determine how sociocultural factors affect these interactions and how they impact the configuration of daily practices and quality of life. Viewing these practices and interactions through a transdisciplinary lens allows for a comprehensive understanding of their complexity. Addressing not only the availability of healthy food, as well as the physical and economic access to it, but also food’s relational, cultural, and social perspective would allow us to face its convoluted phenomenon in a multidimensional, intersectional, and transdisciplinary manner. This new knowledge could result in a better causal diagnosis, an update of the Chilean food environment model, and the proposal of evidence-based national and local public policies to better approach this complex problem.

To improve our understanding of what happens within the food environments, this study aims to explain the practices and interactions of actors in food environments in order to approach the problem of unhealthy eating with a transdisciplinary approach. To reach this aim, this protocol has the following objectives:

1. To describe people’s practices *within* different food environments.
2. To describe people’s interactions *with* different food environments.
3. To analyze the class, gender, and cultural determinants of food environment configurations.
4. To review the Chilean model of food environments, considering interactions, practices, determinants, and transdisciplinarity.
5. To generate recommendations for national and local food and nutrition public policy.

This project aims to answer the following question: How are practices and how do actors interact in food environments? We hypothesize that the practices and interactions of actors in food environments exceed the explanations provided by current theoretical models and require a transdisciplinary approach.

## Methods

### Research Design

This study follows a nonexperimental, cross-sectional, mixed methods study design, which aims to understand the complexity of actors’ practices and interactions in food environments based on the Chilean model [24]. The mixed methods approach will be used for the purposes of triangulation and complementarity. Triangulation is used to visualize the same phenomenon with different methodological perspectives, while complementarity is applied for a broader, deeper, and more comprehensive social understanding of a complex phenomenon [45]. In addition, it will be sequential, so that the quantitative methodology will be used before the qualitative one [45].

The mixed methods approach proposed in this research and the use of ANT as the theoretical orientation enable the integration and transformation of knowledge from different perspectives, blurring disciplinary boundaries. Thus, it will contribute to defining, focusing, exploring, and understanding a complex phenomenon, which is the object of this study [46,47]. Finally, the study will be conducted in 4 phases described as follows:

#### Phase 1: Systematic Literature Review

A systematic literature review is being conducted to update conceptual elements about food environments and provide context for the subsequent phases of the study. The literature review follows the PRISMA (Preferred Reporting Items for Systematic Reviews and Meta-Analyses) protocol [48]. The literature review aims to answer the question: What is new in the evidence of food environment that is not considered in the Chilean Model? To answer this question, we have selected 2 search strategies. First, during 2023, a general search about the food environment was conducted using the terms observed under “general search about food environment” shown in Textbox 1. The terms were searched in English and Spanish. Then, in 2024, a specific search was performed focusing on each food environment included in the Chilean model, using the terms under “search for specific food environments” shown in Textbox 1. In this last search, we also included terms related to the digital food environment and terms related to the influences of food environments.

**Search terms used for the systematic literature review.**

**General search about food environment**

“Food environment,” “eating environment,” “nutrition environment,” “food availability,” “food access,” “social food environment,” “community food environment,” “retail food environment,” “information nutrition environment,” “obesogenic environment,” “unhealthy food environment,” “healthy food environment,” “food shopping,” “unhealthy/healthy food marketing (advertising),” and “food environment typology.”

**Search for specific food environments**

*Home food environment*: “home food environment,” “family and food environment,” and “domestic food environment.”

*Restaurant food environment*: “restaurants and food environment,” “coffee shops and food environment.”

*Institutional food environment*: “organization food environment,” “educational institution (school/university/college/technical school food environment),” and “work food environment.”

*Supply food environment*: “community food environment,” “retail food environment,” “neighborhood food environment,” “urban food environment,” “rural food environment,” “harvest markets and food environments,” “markets and food environments,” “convenience store and food environment,” and “market/convenience store/strip center and food grocery.”

*Street food environment*: “street food environment,” “food truck and food environment,” and “community food environment.”

*Digital food environment*: “digital food environment,” “delivery and food environment.”

*Others*: “women/children/adults and food environment,” “agency and food environment,” “migration and food environment,” “social determinants of health and food environment,” “socioeconomic level and food environment,” “educational level and food environment,” “gender and food environment,” “social class/position and food environment,” “time and food environment,” “urban structure and food environment,” “city and food environment,” “geographic access and food environment,” and “social food environment.”

Papers were included if they were published after 2015 (when the Chilean food environment model was developed); they were in English, Spanish, or Portuguese; they were qualitative, quantitative, or mixed methods studies; systematic reviews; or other review types, meta-analyses, and reports. Papers were characterization or observational studies, all related to humans. Studies related to eating disorders, body image distortion, or specific food topics unrelated to food environments were not included. The search for papers was performed in academic databases such as Web of Science, Scopus, PubMed, SciELO, LILACS, and Cochrane. An additional nonacademic search was conducted in the databases of the World Health Organization, the Pan American Health Organization, and the Food and Agriculture Organization of the United Nations, among others. Finally, a specific search was conducted in journals that publish food environment research. The paper search was conducted from July 2023 to April 2024.

We hired 2 methodologists (DS and AR) and experts in systematic literature review to organize this phase. One methodologist (DS) carried out document identification (phase 1 in the PRISMA protocol). We hired 2 research assistants (LI and CZ) for conducting the document selection (phase 2). Both research assistants independently conducted the paper selection. Phase 1 and phase 2 were conducted using the Rayyan software [49]. Four researchers (the authors of this manuscript) are conducting the data extraction and analysis of the selected documents (phase 3), and they will conduct the data synthesis (phase 4). The 2 methodologists are supervising all the phases. Study quality will be evaluated by the 2 methodologists according to the GRADE (Grading of Recommendations, Assessment, Development, and Evaluation) framework [50].

#### Phase 2: Application of the Nutrition Environment Measure Scale—Perceptions Adapted to Chile Instrument

##### Participants

The participants were adults older than 18 years who performed some or any food purchases for their households. The participants were selected from households in 2 neighborhoods in the urban area of the metropolitan region, selected according to the communes’ level of multidimensional poverty [51]: one with a high level of multidimensional poverty and the other with a low level of multidimensional poverty [52,53].

We excluded people who do not speak Spanish, who have lived in the country for less than 5 years, who have a disability that does not allow them to complete the project activities, or who follow a very restrictive diet either due to disease or by choice, as it may profoundly alter their relationship with the food environment.

For this phase, we expect to obtain people’s perceptions of their food environments. Therefore, it is an exploratory phase. In consequence, to calculate the sample size, we assumed that there was an infinite population for each neighborhood and that the proportions of people living in those communes were unknown; therefore, unknown proportions and an error of 5% were used [54]. Also, we assumed a normal distribution. With this, a sample size of 384 people per neighborhood was calculated, meaning that, for the 2 neighborhoods, a sample of 768 participants was needed.

##### Instrument

In the Chilean food environment model, the domestic food environment is considered the actor’s starting point and is described as one of the most complex environments due to the diversity of households that can be found [25]. It is also the main socialization space where most of the food preferences and traditions are defined, symbolized, transmitted, and reproduced [55]. Based on this context, the experiential work of this study begins with the application of an instrument based on the Nutrition Environment Measure Scale—Perceptions (NEMS-P) questionnaire developed by Green and Glanz [56], which evaluates the perceptions people have about their food environments and the practices they carry out there, starting in the home food environment. NEMS-P has been shown to be an easy-to-understand instrument [56], has good test-retest reliability (Kappa Index > 0.6 for most items), and acceptable internal consistency (Cronbach a values of 0.6-0.7). This instrument has been adapted to the Chilean food environment model and has been validated in this population by part of the team presenting this proposal [57]. The Nutrition Environment Measure Scale—Perceptions adapted to Chile (NEMS-P-Ch) had an acceptable reliability with Cronbach a values between 0.44 and 0.82 for the items [57].

Based on the psychometric analysis and the experience in previous research using the NEMS-P-Ch, we shortened this instrument to adapt it to the recommended time for conducting in-person surveys or questionnaires (25-30 minutes) [58]. This shortened version had 31 questions divided into six sections, similar to the original instrument: (1) home food environment; (2) food supply food environment; (3) eating out food environment (including restaurant and street food environment); (4) institutional or organization food environment (work or study places); (5) self-perception of body weight, health, and food; and (6) general household background. This short version of the NEMS-P-Ch has the same internal consistency as the larger version (data not yet published). Like the original NEMS-P, the shortened version of NEMS-P-Ch includes different types of items: direct response (eg, “On average, how many times a month do you eat in a restaurant?”), multiple choice (eg, “Regarding your meals at this place of study or work, you usually… a. Buy your meals there, b. Shop around this place, c. Bring food from home”), yes/no options (eg, “Could you tell me if any of the following foods were available in your home during the past week? Fruits such as oranges, bananas, apples, pears, peaches”), and Likert-type scales that evaluate the degree of agreement, appropriateness, importance, ease, or frequency (eg, “It is easy to buy fresh fruits and vegetables in my neighborhood: never, occasionally, almost always, always”).

##### Data Collection

The Microdata Center at the Universidad de Chile conducted the data collection. This center specializes in conducting national, regional, and local surveys [59]. Based on their previous experiences in survey research, they randomly selected 102 blocks in both neighborhoods: 50 from the low socioeconomic neighborhood and 52 from the high one. In each of these blocks, a trained team member did the census registration of all the addresses corresponding to homes in a database. From this list of addresses in the database, they randomly selected homes to visit. They randomly selected 12 homes per block in the low socioeconomic neighborhood and 14 in the high one. In addition, to ensure that they completed the sample size, they randomly selected 36% and 47% more homes in the low and high socioeconomic–level neighborhoods, respectively.

We trained 20 interviewers. These interviewers had experience in survey application and had worked for the Microdata Center. We conducted a 4-hour training program that included ethics (including consent process) and a question-by-question review and revision. In addition, we prepared a survey manual for the interviewers, which was sent to them by email before the training. This group of trained interviewers applied the NEMS-P-Ch in person from October to December 2023, visiting the selected homes in each neighborhood.

##### Data Analysis

Descriptive statistics will be applied for each of the scales included in the instrument. Items that describe food environments or people’s interactions with them were scored. Negative scores are given for less healthy food environments, while positive scores are given for the opposite. The perception of the environments between households in districts with different levels of multidimensional poverty will be explored by comparing the scores using the *t* test or the Wilcoxon-Mann-Whitney test, depending on whether the data meet the normality assumption [60]. We will also consider adjusting a multiple linear regression model for each of the perception scales, using the scale score as the response and the level of multidimensional poverty as a variable, controlling for other individual characteristics such as age, gender, and others [61]. Statistical analyses will be performed with SPSS (version 29.0; IBM Corp) software.

#### Phase 3: Conducting Focus Groups (2024-2025)

##### Participants and Sampling

The participants will be adults aged 18 years or older who (ideally) participated in phase 2. The sample determined will be by convenience. Participants who answered the NEMS-P-Ch by email or phone will be invited to participate in one of the focus groups. If we cannot complete the necessary sample for each focus group with the participants from phase 2, we will invite other people who meet the inclusion criteria from the corresponding neighborhood. We will ask for the collaboration of community members from the included neighborhood to invite other participants.

##### Data Collection

In this intermediate phase, we will conduct focus groups that are considered workshops with the actors for listening-conversation-reflection. Focus groups are a technique that aims to produce a set of social conversations based on group discussion of relatively focused topics [62]. The focus groups will analyze and interpret how actors’ meanings develop in their food environments and what the significant associations are, thus accessing the knowledge and perceptions that guide their decisions, choices, and actions, which leads to their configuration [62]. The focus group will also allow us to understand how food environments determine food behaviors from the point of view of those who interact in these environments. In this manner, we will be able to obtain, as ANT points out, *their* theories about what makes up the social component. The generation of the focus groups and the creation of the questions will be based on the results of phase 2 and on the background information emerging from the systematic literature review (phase 1).

A total of six focus groups will be carried out for each neighborhood participating in phase 2, distributed as follows: (1) a group with people who have a paid job, (2) a group with people without a paid job, (3) a group with Chileans only, and (4) a group with non-Chileans. Finally, there will be a group of (5) only men and (6) only women.

The focus groups will be conducted by trained personnel. The duration of each focus group will be a maximum of 1.5 hours (90 minutes), and between 5 and 8 people will be invited. Each focus group will be recorded and transcribed.

##### Data Analysis

The focus group recordings will be transcribed and reviewed by a team member to ensure the accuracy of the transcription. The information will be analyzed in accordance with the principles of the Grounded Theory of Strauss [62], which establishes three main moments: (1) *open coding*, whose main objective is to discover and develop a conceptualization that emerges from the data. This cellular approach to information, rather than global, reduces complexity and synthesizes the information from the construction of codes, which will be consistent with the research objectives; (2) *axial coding*, which articulates networks in order to relate concepts and analytical categories, establishing hierarchies with subcategories—properties and dimensions—around a category taken as the axis and which seeks to create schemes of understanding to advance toward analytical resolutions over descriptive ones; and (3) *final integration of findings*, which refers to the presentation of tentative explanations—some call this phase *selective coding* [63]. Atlas.ti (version 24; Cleverbridge GmbH) software will be used to provide technological support in the systematization of the large amount of information collected.

#### Phase 4: Ethnography (2025-2026)

##### Participants

The participants will be adults aged 18 years or older who (ideally) participated in phase 2 or 3 of this proposal. The sample for this phase will be selected according to the cases’ representativeness and relevance. We will create profiles of participants (case studies) based on the findings of the previous phases. We will select subjects associated with characteristics and life conditions that stand out as distinctive and then select the determinant features of these subjects, that is, those with attributes that reflect meanings, decisions, and behaviors that are of interest for an in-depth study or situated knowledge to enhance the analysis of particular cases. Each profile will offer us an exhaustive and qualitative description of a specific situation or condition that not only involves a detailed understanding of what we intend to study but also provides the possibility of considering new aspects that may help us develop more relevant concepts. Likewise, it will allow us to identify key elements and variables that have an impact and that show the links between them, providing the phenomenon with an explanation that includes relationships, variability, and complexities to achieve an approach between the theories included in the theoretical framework and the reality under study.

##### Data Collection

###### Overview

Once the previous phases have been carried out, we will address the main principle of the ANT, which proposes “following the actors themselves” in order to trace the sites of production of interactions [31]. Through this ethnographic method, we can directly and empirically explore the actors’ daily interaction with the food environments they operate in by using different sources and techniques combined to carry out a “field experience” and thus access the meanings and senses in a nondisruptive and contextualized manner [59]. For this purpose, the following will be carried out.

###### Participant or Ethnographic Observation

This technique refers not only to the act of looking but also to the act of observing critically and attentively [64]. It focuses on exploring the daily contexts in which the subjects of study develop by means of an in situ and systematic inquiry, in which the researcher is partially inserted. That is, he or she participates as an observer who acts as an external agent to the context being studied precisely to be able to capture the dimension of daily routines and practices [64]. Field notes and photographs will be taken by the research team.

###### In-Depth Interviews

We will use a conversational technique that is open, flexible, and reflexive, enabling a trusting and horizontal dialogue between researcher and interviewee [65]. A guideline of questions will be created and constantly reviewed for cultural appropriateness and questioning capacity in relation to the aspects of interest. Interviews will be conducted where people deem convenient, provided that these places are associated with the food environments they experience, such as the domestic and supply. These interviews will be audio recorded.

###### Ethnography

We will conduct 15 case studies, following Eisenhardt’s principles, which state that it is possible to generate theorization between 4 and 10 cases [66]. The ethnography will be conducted for 4-5 hours, in 2-3 days (depending on the participant’s availability), and especially in those moments when the participant is relating with food and food environments such as grocery shopping, cooking, commuting, among others. The ethnography will be conducted by trained personnel.

##### Data Analysis

Team members will transcribe the interview. A team member will review this transcription to ensure the accuracy. Field notes will be transcribed into a Word document. The qualitative data analysis will be carried out using Atlas.ti (version 24) software, following the Grounded Theory approach described before [63,67].

### Triangulation and Complementarity of the Obtained Data

As a merged construction of the methodological process, triangulation will be carried out to understand the phenomenon with the use of data of a different nature whose priority is to compare [68] (1) level of complementation of relevant information to group dimensions and meanings; (2) levels of convergence of information to agglutinate common dimensions and generate categories and codes; and (3) levels of divergence of information to segment dimensions of meanings.

On the other hand, there will be elements of the quantitative data that will be complemented and deepened with the speeches and explanations found in the qualitative phase [45]. In other words, qualitative data will provide information that has not been collected by quantitative methodologies. We will be able to inquire about the “*why*” and the “*how*” of the visualized phenomenon, allowing a better understanding of it. In this sense, it is mainly in the data analysis where the mixture of methods will be carried out. In addition, through the qualitative information, we will be able to apply the ANT principles to understand the interactions between human and nonhuman actors that influence their decisions made in food environments (which will probably also appear in the quantitative data). In consequence, we will create the network of actants in food environments.

The information obtained through the triangulation and complementarity of the data, as well as ANT, will be useful to understand how actants and food environments cocreate everyday practices. It will thus enable us to create a practical model of the practices and interactions of actors in their food environments, reflecting the transdisciplinary approach of the study.

### Ethical Considerations

This protocol was accepted on January 2023 and funded on June 1, 2023. Ethical approval was obtained on May 27, 2023, from the Ethics Committee of the College of Medicine, Universidad de Chile (#013-2023). Informed consent is obtained from all participants before the beginning of each study phase after explaining the activities they will participate in and their rights and duties. All participant data will be de-identified, and only aggregated group data will be presented in all reports of the study findings. Participants in phase 2 did not receive any compensation. Participants in phase 3 will receive transportation compensation of about US $10 (10,000 Chilean pesos), while participants in phase 4 will receive US $50 (50,000 Chilean pesos) for their time in the ethnography activities.

## Results

### Advances in Phase 1: Systematic Literature Review

For the general search, we retrieved 712 documents. For the specific search about each food environment in the Chilean Model, we retrieved 773 documents. As of May 2024, a total of 109 documents from both searches are in the data extraction phase. We will finish the systematic literature review by August 2024. The results are expected to be submitted for publication by the end of 2024.

### Advances in Phase 2: Application of the NEMS-P-Ch Instrument

We applied the instrument to 388 participants in the low socioeconomic–level neighborhood and 397 from the high socioeconomic–level neighborhood, reaching a total sample of 785 participants. Participants have a median age of 51 years, with a minimum of 18 years and a maximum of 92 years. Participants from the low socioeconomic neighborhood were older than their counterparts (55 vs 47 years; *P*=.003).

Most of the participants were female (460/785, 58.6%), and 0.1% (1/785) perceived themselves as other gender. No differences between neighborhoods were found. Data analysis is planned to be finished by July 2024, and the first results are expected to be submitted for publication by the end of 2024.

### Advances in Phase 3

Phase 3 started in July 2024 and will be finished by September 2024. By the end of July, 6 focus groups have been conducted: the groups of women, men, and nonworkers in both neighborhoods. Between 5 and 11 participants took part in the focus groups. Pending focus groups are planned for August and September 2024.

### Pending Phase and Activities

Phase 4 will take place from November 2024 until September 2025. Data triangulation and integrative data analysis will occur from September 2025 to January 2026.

## Discussion

### Principal Findings

This study will examine what happens within food environments from a transdisciplinary perspective. Through its innovative feature, it will generate new experiential and practical knowledge of how the actors use, interact, and mobilize in their food environments and, in doing so, characterize the actors’ food practices. We expect to find new elements of food environments that have not been described before and complex interactions between actors in these environments. These interactions go beyond a simple and traditional explanation of food practices. All this new knowledge will be translated into a new framework of food environments that will include updated information about the phenomenon and an integrated approach that allows an understanding of how people make decisions in food-related processes [33].

Chile has some definitions and concepts that allow a theoretical understanding of food environments as probable agents of poor nutrition in the population [25]. However, it is necessary to complement this theory by studying people’s daily experiences, decisions, and interactions with their environments, an aspect not previously studied that can also serve as a model for other countries.

The study will be carried out using mixed methods, allowing a deeper investigation of the interaction and configuration of food environments, delving into the “why” and “how” of the visualized phenomenon. For Turner et al [33], using mixed methods research has the potential to fill the gap in our understanding of people’s interaction with food environments. Previous studies have used mixed methods in food environment research [69-73]. However, these studies included exploring 1 food environment (most commonly, the retail food environment). This study will give information about the 5 food environments included in the Chilean model: home, organizational, street, restaurant, and supply food environments [25].

The use of ANT as a framework is also an innovation in the study of food environments. The complexity of this research problem forces us to look for answers through methodologies that go beyond fragmentary research, moving from linear observation to interactional observation. The ANT will allow us to innovate in studying the relationship between food environments and the actors (human and not human) moving inside and through them, turning this new information into an understandable framework [74].

Transdisciplinarity reorients scientific work and forces the integration of different areas of knowledge, promoting a symmetrical and dynamic relationship of perspectives that enrich traditional approaches and are projected through a prism, giving rise to new and innovative perspectives [75]. In this case, a dialogue between different medical disciplines and social science is assumed. Transdisciplinarity challenges us to a joint conceptual construction of the study problem, objectives, methodologies, analysis, and proposals. This type of research paves the way for the necessary and different points of view, allowing for the multiprofessional and intersector approach that this complex problem requires.

Despite the study’s relevance and information quality, we acknowledge some limitations. First, this study will be conducted only in an urban region of Chile, leaving out other relevant elements from rural regions. In addition, our study does not include geographic information as most mixed methods research in food environments [69,73]. Finally, we will focus exclusively on the adult population residing in 2 neighborhoods within a single region of the country. Although these elements could enrich the understanding of the food environment, our study will represent an integral view of people’s food practices and decisions from their home food environment to other environments where they move daily.

The results of this study will be disseminated through peer-reviewed publications in Web of Science journals and through abstracts, posters, and presentations at national and international conferences. The results will also be reported to local and national governments to inform policies. We will send infographics with the results to participants at each project phase. Finally, the study findings will be posted on the Transdisciplinary Group for Obesity of Populations website at the Universidad de Chile (GTOP-UChile) of which the authors are part [76].

### Conclusions

This study will provide a holistic view of food environments. This study will present an integrated view of food environments and their configuration versus the fragmented view of the current Chilean model. In this context, this study will also open new challenges and lines of research on this subject. The new information shown in this study will help develop evidence-based and more comprehensive cross-sectoral public policies and create a new framework for food environments with an interactive approach between individuals and the environment. Like the previous Chilean model of food environments, this new one could be used to study food environments in other Latin American countries [75,77,78].

## Appendix

Multimedia Appendix 1 Peer-review report by the Concurso Nacional de Proyectos FONDECYT Regular 2023 - Agencia Nacional de Investigación y Desarrollo (Chile).

## Abbreviations

ANT: Actor-Network Theory
GBD: Global Burden of Diseases, Injuries, and Risk Factors Study
GRADE: Grading of Recommendations, Assessment, Development, and Evaluation
GTOP-UChile: Group for Obesity of Populations website at the Universidad de Chile
NEMS-P-Ch: Nutrition Environment Measure Scale—Perceptions adapted to Chile
PRISMA: Preferred Reporting Items for Systematic Reviews and Meta-Analyses

## Data Availability

The data generated from this study will be made accessible in the future to facilitate collaborative research inquiries. Requests for access must be formally approved by the principal investigators.
