# Supplementary material for: Rethinking the Social Determination of Food in Chile Through Practices and Interactions of Actors in Food Environments: Nonexperimental, Cross-Sectional Study
Source: JMIR Res Protoc. 2024 Sep 13;13:e62765. doi: 10.2196/62765 (PMC11437218; doi:10.2196/62765)
Supplement: Multimedia Appendix 1 [file resprot_v13i1e62765_app1.pdf]

ANID/Subdirección de Proyectos/N°454

Santiago, 7 de Marzo del 2023
Ref.: Proyecto Nº 1230545

Señora
PATRICIA ANDREA GALVEZ ESPINOZA

Estimada señora GALVEZ:

En nombre de la Agencia Nacional de Investigación y Desarrollo, me dirijo a usted para informar que ha sido aprobado su proyecto N°1230545, postulado al Concurso Nacional de Proyectos FONDECYT Regular 2023.

En la presente convocatoria, concursaron 1.873 proyectos, de los cuales 695 (37,1%) fueron adjudicados. Su proyecto fue evaluado en el Grupo de INTERDISCIPLINARIO Y TRANSDISCIPLINARIO, donde obtuvo el lugar N° 23 y un puntaje de 4,370. En este Grupo concursaron 111 propuestas, evaluaron 84, adjudicaron 40 (36%) y la calificación del último proyecto financiado fue de 4,160 puntos.

A continuación de esta carta, se informan las calificaciones y comentarios que recibió el proyecto y se incluye un certificado que acredita su adjudicación. Asimismo, en el Sistema de Evaluación en Línea, podrá acceder:

a) Al presupuesto aprobado para cada año de ejecución. Recuerde que puede redistribuir los fondos anualmente, si lo estima pertinente.
b) Al Informe Autorizaciones-Certificaciones donde se indica los documentos que deberá presentar al inicio del proyecto, si corresponde.
c) Al botón “Decisión de Ejecutar”, donde deberá comunicar si acepta o rechaza la adjudicación del proyecto en un plazo máximo de 10 días hábiles administrativos, a partir de la fecha de esta carta.

Respecto al proceso de Firma de Convenio y Transferencia de recursos, revise el instructivo correspondiente, disponible en InstructivoFirmaConvenioRegular2023.pdf. En caso de consultas, debe realizarlas a través de Ayuda ANID (https://ayuda.anid.cl).

En la página web de ANID, Subdirección de Proyectos de Investigación, encontrará información respecto al proceso de evaluación del Concurso.

Desde ya reciba nuestras felicitaciones por este importante logro en su carrera científica.

Saluda atentamente a Ud.,

ALEJANDRA VIDALES CARMONA
Subdirectora
Subdirección de Proyectos de Investigación
Agencia Nacional de Investigación y Desarrollo

RESULTADO DEL PROCESO DE EVALUACIÓN

CALIFICACIONES OBTENIDAS POR EL PROYECTO Nº 1230545

**Factores de evaluación / Ponderación**	**Calificación**
Etapa 1:<br>Productividad del/de la Investigador/a Responsable (30%) (*)	5,0
Etapa 2:<br>Calidad, Factibilidad y Novedad Científica o Tecnológica (70%)	4,1

(*) información respecto a la calificación obtenida en este ítem, será enviada en una carta al correo electrónico informado en su postulación.

COMENTARIOS DE LA EVALUACIÓN
PROYECTO Nº 1230545

Nota:
Los comentarios que emita el Grupo de Evaluación podrá relevar aspectos que considere necesario transmitir al/a la postulante, de acuerdo a numeral 9.5 de las bases concursales.

CALIDAD, FACTIBILIDAD Y NOVEDAD CIENTÍFICA O TECNOLÓGICA DE LA PROPUESTA

Prácticas e interacción de actores en ambientes alimentarios: repensando los determinantes sociales de la alimentación.
IR: Patricia Galvez (U de Chile). Co-I: Daniel Egaña, Lorena Rodríguez y Carolina Franch

Existe un creciente problema de malnutrición por exceso en la población chilena, la que según la ENS del 2016-17 llega al 74% a nivel nacional. Este problema se caracteriza por una alta prevalencia de sedentarismo y una alta exposición a alimentos ultra procesados, los que afectan múltiples áreas de la vida, incluyendo salud y calidad de vida. Los comportamientos alimentarios y de actividades física son complejos y dependen de la interacción de múltiple elementos y nivel (factores individuales, proximales, y estructurales). En la actualidad se han conceptualizado como ambientes obesogénicos aquello que favorecen condiciones y oportunidades que promueven prácticas poco saludables.

Respecto a los ambientes alimentarios, existen diversos modelos, aunque los autores parecieran adherir a una de las definiciones más recientes (2017) que dice que son “los contextos físicos, económico políticas y socioculturales que enmarcan las interacciones que tienen los consumidores con el sistema alimentarios para la adquisición, preparación y consumo de alimentos”. Parte de esto se recoge en un documento elaborado por parte del equipo de investigación para el MINSAL en la que identifican y definen para Chile 5 ambientes alimentarios: domestico, institucional y organizacional, vía pública, restauración y abastecimiento. Este modelo

es el que se usó para el desarrollo de la Política Nacional de Alimentación y Nutrición y la estrategia Nacional de Salud que establece los objetivos sanitarios para la década 2030.

Los autores mencionan que, pese a que se ha generado evidencia respecto a los ambientes alimentarios en Chile, aun es insuficiente para comprender la forma en que los ambientes determinan la salud nutricional de las personas. Particularmente, los investigadores están interesados en comprender el cómo ocurren las interacciones persona-ambiente alimentarios y en cómo estos interactúan entre sí. Al respecto, los autores describen la Teoría del Actor-Red (ANT en inglés) como un modelo que permitiría avanzar en ese objetivo. ANT enfatiza el carácter interactivo, transformativo y creativo de los "actantes" (personas o no personas), los que se relacionan de forma no mecánica ni lineal.

Usando este marco conceptual lo investigadores se plantean la pregunta "¿cómo son las prácticas y cómo interactúan los actores en los ambientes alimentarios?" La hipótesis que explicitan es que "las prácticas e interacciones de los actores en los ambientes alimentarios excede las explicaciones que provienen de los modelos teóricos actuales y requieren de una aproximación transdisciplinaria". En este punto surge la duda respecto a si esta debiera ser la hipótesis de una investigación empírica. Buena parte del desarrollo del marco teórico de este proyecto se enfoca en justificar la necesidad de un enfoque transdiciplinario, por lo que no parece coherente plantearlo como una hipótesis de investigación. De hecho, en sus objetivos proponen desarrollar una investigación de tipo interdisciplinario, por lo tanto, ya estarían confirmando su hipótesis.

El objetivo general del proyecto es "explicar las prácticas e interacciones de los actores en ambientes alimentarios, para aproximarse al problema de la alimentación no saludables desde una aproximación transdisciplinaria".

Plantea 5 objetivos específicos:
1. Describir las prácticas de las personas dentro de diferentes ambientes alimentarios
2. Describir las interacciones de las personas con diferentes ambientes alimentarios
3. Analizar los determinantes clase, género y cultura de las diferentes configuraciones de ambientes alimentarios
4. Revisar el modelo de ambientes alimentarios chileno considerando interacciones, prácticas, determinantes y transdisciplinariedad
5. Generar recomendaciones de políticas pública sobre alimentación y nutrición a nivel nacional y local

Para esto el equipo de investigación plantea desarrollar 4 etapas secuenciales, definiendo previamente criterios de inclusión (18+ años, de zonas urbanas de la RM) y exclusión (que no hablen español, <5 años de residencia en el país, participantes que por motivos físicos o cognitivos no puedan realizar las actividades del proyecto o que por decisión o enfermedad tengan una relación alterada con sus ambientes alimentarios) de participantes. A nuestro juicio, no queda claro por qué excluir a personas que hayan llegado a Chile hace menos de 5 años, especialmente en el contexto migratorio actual.

• Fase 1. Revisión de literatura en bases de datos científicas y páginas web de organismos especializados (OMS, OPS, FAO, entre otras.).
• Fase 2. Cuantitativa, que consiste en aplicar el instrumento NEMS-P, cuya adaptación y validación en Chile realizaron los propios investigadores de esta propuesta en un proyecto FONIS con resultados no publicados aún. El instrumento evalúa la percepción que tienen las personas respectos a sus ambientes alimentarios y las

prácticas que desarrollan en ellos. Considera diferentes secciones, incluyendo percepción y hábitos alimentarios y autopercepción del peso corporal y salud. Sin embargo, las secciones sobre ambientes alimentarios no se condicen 1 a 1 con los ambientes alimentarios propuestos en el modelo chileno (Cerda y cols. 2016) ni menos con la definición más amplia mencionada en los antecedentes, faltando, por ejemplo, el ambiente institucional/organizacional. El análisis de esta fase considera la comparación de puntajes entre personas de diferentes niveles de pobreza multidimensional. El n propuesto son 1190 personas con selección aleatoria simple (¿personas? ¿viviendas?), no obstante, no se menciona qué marco muestra se utilizará (¿MMM2016?)
• Fase 3. Cualitativa a través de grupos focales. Se propone realizar 6 grupos focales de 5 a 8 personas, con grupos heterogéneos, estratificando por nivel de educación, nacionalidad y sexo.
• Fase 4. Cualitativa a través de etnografía. Esta fase contempla dos componentes: 1) la observación etnográfica para conocer rutinas diarias y prácticas en el contexto mismo en el cual las personas se desenvuelven naturalmente. Acá no menciona duración ni define claramente una estrategia de inserción en la vida cotidiana de estas personas. 2) Entrevistas en profundidad de hasta 90 minutos. Para esta cuarta fase se consideran 15 personas.

Posterior a la realización de estas 4 fases se realizará un proceso de triangulación de la información para analizar los niveles de completitud, convergencia y divergencia de la información recogida

El proyecto es de 3 años, con actividades coherentes con las descritas en la metodología. El equipo de investigación tiene experiencia en el desarrollo de investigación sobre ambientes y comportamiento alimentarios. Se menciona la colaboración del equipo en dos proyectos FONIS relacionados a la temática y la IR está finalizando un proyecto Fondecyt de Iniciación sobre influencias familiares en la dieta y estado nutricional de mujeres de bajos ingresos. Los recursos solicitados están en el tope establecido por Fondecyt y son coherentes con las actividades propuestas. La mayor cantidad de recursos está asignada a gastos operacionales y personal técnico. Contempla 1 estudiante de postgrados y 3 de pregrado por año a contar del año 2.

El proyecto está bien escrito, describe modelos conceptuales claros sobre una temática relevante para el país. El equipo tiene experiencia, historial de colaboración en proyectos y publicaciones científicas. El proyecto se plantea como transdisciplinario, y efectivamente no se distinguen los bordes disciplinarios, integrando aspectos conceptuales y metodológicos de la de nutrición, antropología, psicología, sociología y salud pública. El estudio se realizará con métodos mixtos, lo que permitirá profundizar en el fenómeno de la interacción y configuración de los entornos alimentarios, profundizando en el "por qué" y "cómo" del fenómeno visualizado.

Hay aspectos, sin embargo, que son poco claros y que, a nuestro juicio, no dan cuenta de la complejidad del problema tal y como se describen en los antecedentes del proyecto. Por ejemplo, el ambiente alimentario institucional/organizaciones pareciera que no está incluido en el instrumento propuesto en la fase cuantitativa, aunque tampoco se explica en detalles ni se incluye a modo de anexo el instrumento a utilizar. Además, el comportamiento psicométrico del instrumento tampoco está claro, y solo se dice que es similar al de la versión original, la que a su vez presenta valores moderados de consistencia interna y confiabilidad test-retest. Proponen dos objetivos específicos (el 4 y el 5) que no son objetivos de investigación, sino actividades o productos de la investigación. Se menciona a lo largo de todo el proyecto (incluyendo título y objetivos) los térmicos “prácticas” e “interacción”, pero no hay una definición conceptual ni operativa de ellos. Por ejemplo, ¿Las prácticas se refiere a hábitos alimentarios? ¿Incluye las prácticas culinarias? ¿Qué otros aspectos consideran? Tampoco queda claro cómo se recoge el modelo ANT y toda su complejidad en el desarrollo de la

investigación. En el objetivo específico 3 se menciona la palabra "cultura" como un determinante de los ambientes alimentarios, pero no queda definido cómo se analizará eso. Tampoco hay una justificación respecto a la delimitación del espacio geográfico que cubre la investigación. ¿Por qué solo considera zonas urbanas de la Región Metropolitana? Pareciera, por ejemplo, que las zonas rurales donde se concentra casi la totalidad de la producción agrícola es un espacio relevante de considerar. De igual forma, hay una importante heterogeneidad territorial y cultural en Chile, por lo que limitar la investigación a la Región Metropolitana limitaría la generabilidad de los resultados a otras regiones y contextos del país. Por último, la bibliografía es relativamente antigua, donde solo 7 de las 77 referencias son de 2021-22.

Finalmente, hay aspectos formales que pudieran mejorarse, por ejemplo, dividir el texto en subsecciones, destacar ideas centrales, mejorar la consistencia entre pregunta, hipótesis objetivas general y objetivos específicos.

CERTIFICADO ADJUDICACIÓN
CONCURSO FONDECYT REGULAR

7 de Marzo del 2023

Alejandra Vidales Carmona, Subdirectora, Subdirección de Proyectos de Investigación, de la Agencia Nacional de Investigación y Desarrollo, certifica que Doña PATRICIA ANDREA GALVEZ ESPINOZA, ha adjudicado el proyecto N°1230545 en el Concurso Nacional de Proyectos FONDECYT Regular 2023, titulado PRACTICES AND INTERACTIONS OF ACTORS IN FOOD ENVIRONMENTS: RETHINKING THE SOCIAL DETERMINATION OF FOOD.

El proyecto, patrocinado por el/la UNIVERSIDAD DE CHILE, contempla una duración de 3 años – desde 1 de Abril del 2023 hasta el 31 de Marzo del 2026 .

Se extiende el presente certificado al/a la interesado/a, para los fines que estime conveniente.

Alejandra Vidales Carmona
Subdirectora
Subdirección de Proyectos de Investigación
Agencia Nacional de Investigación y Desarrollo
